# X-ray crystal structures of the cannabinoid synthases CBCAS, CBDAS and THCAS

**DOI:** 10.1016/j.crstbi.2026.100197

**Published:** 2026-07-16

**Authors:** Jack Domenech, Andrew King, Edward Byrne, Jared Cartwright, Gideon Grogan

**Affiliations:** a Department of Chemistry, University of York, Heslington, York, YO10 5DD, UK; b Jazz Pharmaceuticals, Kent Science Park, Sittingbourne, ME8 9AG, UK; c Department of Biology, University of York, Heslington, York, YO10 5DD, UK

**Keywords:** Biocatalysis, Cannabinoids, Cannabinoid synthases, Berberine bridge enzyme, X-ray crystallography

## Abstract

The enzymes Cannabichromenic Acid Synthase (CBCAS), Cannabidiolic Acid Synthase (CBDAS) and Tetrahydrocannabinolic Acid Synthase (THCAS) are together the major cannabinoid synthase enzymes responsible for the biosynthesis of their respective metabolites from a common precursor Cannabigerolic Acid (CBGA). As the catalysts responsible for generating biological molecules of significant pharmaceutical value, there has been considerable interest in the enzymes with respect to heterologous production, mechanism, and incorporation into synthetic biology pathways for the facile industrial production of these molecules. The enzymes share high degrees of homology, and therefore their distinct specificities are governed by very subtle differences in sequence and therefore structure, although, until now, only a structure for THCAS has been reported. In this report, we present structures of CBCAS, CBDAS and a structure of THCAS at a higher resolution than the known structure, each in complex with their flavin coenzyme FAD. The structures reveal active site differences that may be responsible for the complementary activities observed, in terms of both first-shell amino acid substitutions, but also in more remote residues that influence active site topology through referred effects, or that have effects on substrate access. The structures provide a useful and informative platform for the rational engineering of improved or altered chemoselectivity in these enzymes.

## Highlights

• CBCAS, CBDAS and THCAS has been expressed in *Komagataella phaffii*.
• Each enzyme has been purified using NiNTA, CIEX and SEC methods.
• The crystal structures of CBCAS, CBDAS and THCAS are presented.
• Structures illuminate differences responsible for contrasting chemoselectivities.

## Graphical abstract

**Figure:**
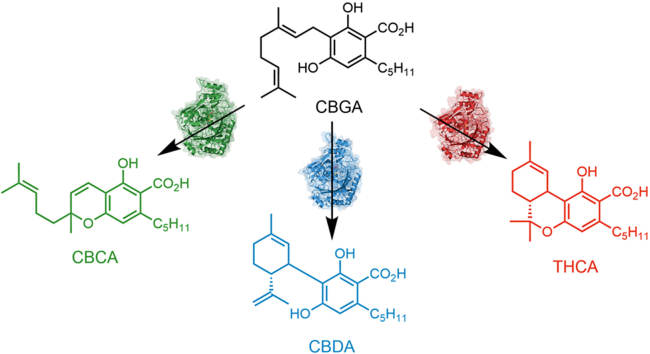

## 1 Introduction

**Fig. 1:**
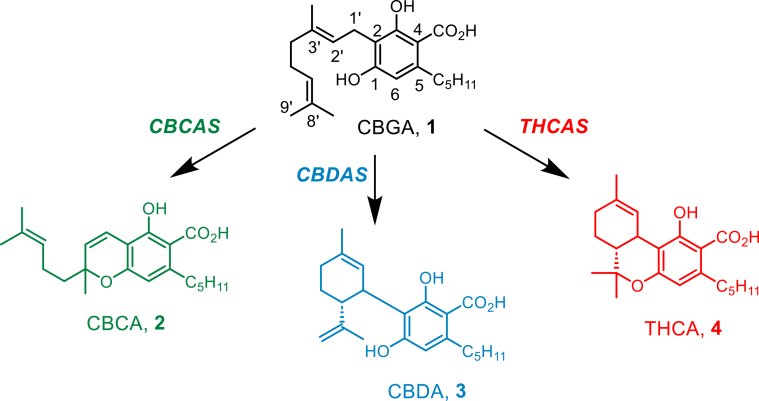
Reactions catalyzed by cannabichromenic acid synthase (CBCAS), cannabidiolic acid synthase (CBDAS) and Δ^9^-tetrahydrocannabidiolic acid synthase (THCAS).

The cannabinoids are a class of plant-derived compounds with a broad spectrum of biological activity (Duczmal et al., 2024). Although a large number (>100) of structurally different compounds have been shown to be present in *Cannabis sativa* (Filipiuc et al., 2021), the major compounds of interest are cannabichromene (CBC), cannabidiol (CBD) and Δ^9^-tetrahydrocannabidiol (THC) each of which is synthesised in the plant from a common precursor cannabigerolic acid (CBGA, **1**, Fig. 1) (Tahir et al., 2021) as its carboxylic acid counterpart (cannabichromenic acid CBCA **2**, cannabidiolic acid CBDA **3** and Δ^9^-tetrahydrocannabidiolic acid THCA **4**, respectively) by predominantly three identified cannabinoid synthase (CSase) enzymes, CBCAS (Laverty et al., 2019), CBDAS (Taura et al., 2007) and THCAS (Taura et al., 1995).

Each of these enzymes is an FAD dependent oxidase of the Berberine Bridge Enzyme (BBE) family (Daniel et al., 2017). There has been considerable interest in these synthases, as they each have current or future potential as biocatalysts for the synthesis of molecules of potent biological activity that are indicated for conditions including seizures associated with Lennox-Gastaut syndrome, Dravet syndrome and tuberous sclerosis complex (CBD, Epidiolex® (Georgieva et al., 2023)) and nausea and vomiting associated with cancer chemotherapy (THC, dronabinol (Bajtel et al., 2022)). Hence, previous research has focused on the heterologous expression of the enzymes, but also on their catalytic characteristics and their incorporation into synthetic biology pathways for the facile industrial synthesis of cannabinoid precursors (Luo et al., 2019; Zirpel et al., 2015; Kosalková et al., 2023). The synthases are closely related in sequence (SI Figure S1), but also display imperfect chemoselectivity in the production of their titular metabolites from CBGA **1**. For example, THCAS synthesises up to 5% of CBDA, and this proportion has been shown to be increased at elevated pH (Zirpel et al., 2018). CBDAS synthesises up to 5% of both CBCA but also THCA (Zirpel et al., 2018). The specificity of the latter enzyme is of special interest, as, although CBD is a molecule with interesting pharmaceutical potential, it does not have the psychoactive properties of its co-product THC. However, as all CBDAS-mediated syntheses of CBDA also produce THCA, this presents significant problems because of the nature of THC as a controlled substance. Hence, understanding the control of selectivity in the cannabinoid synthases would be a worthwhile goal that could be deployed for the selective synthesis of, for example, CBDA with no production of THCA. Some engineering experiments, based on AlphaFold models of the enzymes, have already been reported (Thomas and Kayser, 2023; Dai et al., 2024; Qi et al., 2025). However, for successful engineering experiments, a thorough understanding of the different mechanisms underlying the synthesis of THCA, CBCA and CBDA by the synthases would be required. In 2012, Shoyama and co-workers reported the only extant structure of a cannabinoid synthase, that of THCAS (PDB code 3VTE), refined to a resolution of 2.75 Å and complexed with FAD but neither CBGA nor THCA (Shoyama et al., 2012). The description of the active site thus permitted some speculation about a possible mechanism for THCAS, but not for the distinct selectivities of the homologs, for which no structures have yet been presented. In this report, we present the first structures of both CBCAS and CBDAS, refined to resolutions of 2.43 and 1.70 Å respectively, and also a structure of THCAS at a higher resolution of 2.33 Å. Each is complexed with FAD and, while no CBGA or product complexes were successfully obtained, the structures provide important new information on the differences in active site topology of the enzymes engendered by first shell substitutions, but also more remote effects, and provide a valuable platform for revisiting the engineering of the enzymes for improved or altered specificity.

## 2 Materials and Methods

### 2.1 Gene cloning

The genes encoding CBCAS, CBDAS and THCAS, codon optimised for *K. phaffii*, were ordered from Geneart® (Thermofisher). The genes, lacking the respective 28 amino acid signal peptides, were amplified by PCR using the KOD Hot-Start polymerase kit (Sigma) according to the manufacturer’s instructions, with the addition of an enhancer (0.5 M Betaine, 1.3% (v/v) DMSO) using the primers listed in Table 1. The purchased vector encoding the genes was used as a template for PCRs (10 ng).

**Table 1:** Oligonucleotide primers used to create *K. phaffii* expression vectors.

Primer name	Sequence
CBCAS-F	CATCACCACCACCACAACCCTCAAGAGAACTTCCTGAAG
CBCAS-R	GAGTTTTTGTTCTAGTTAGTGATGTCTTGGTGGCAATGG
CBDAS-F	CATCACCACCACCACAACCCTCGTGAGAACTTCTTGAAG
CBDAS-R	GAGTTTTTGTTCTAGTTAGTGTCTGTGTCTTGGCAGTG
THCAS-F	CATCACCACCACCACAACCCTCGTGAGAACTTCTTGAAG
THCAS-R	GAGTTTTTGTTCTAGTTAGTGATGATGTGGTGGCAATGG
pPICZα-F	CTAGAACAAAAACTCATCTCAGAAG
pPICZα-R	GTGGTGGTGGTGATGATGG

For PCR, the initial denaturation step was run for 5 min at 95°C; denaturation was run for 20 s at 95°C; annealing was run for 10 s at 51.3°C and elongation for 35 s at 70°C; 20 cycles were employed. Amplicons were subject to overnight DpnI digestion following the manufacturer's instructions, and then gel purified using the QIAquick gel extraction kit (QIAGEN).

The expression vector, a modified pPICZα containing a N-terminal His tag immediately downstream of the α signal peptide, was linearised by PCR using the KOD Hot-Start polymerase kit (Sigma) as above, using the primers listed in Table 1. The initial denaturation step was run for 5 min at 95°C; denaturation was run for 1 min at 95°C; annealing was run for 45 s at 58.3°C and elongation for 3.5 min at 72°C; 30 cycles were employed. A final elongation step was used at 72°C for 4 min. Vector DNA was used to template reactions (10 ng).

For each gene, an InFusion HD reaction (Takara Bio) was set up with a 2:1 vector: insert molar ratio as manufacturers instruction, before being used to transform Stellar™ Competent cells of *E. coli* (Takara Bio) by heat shock. The resulting transformants were spread onto low salt LB agar plates supplemented with 25 μg mL^−1^ Zeocin™ and incubated overnight at 37°C. Positive clones for each gene were used to inoculate 10 mL low salt LB medium containing 25 μg mL^−1^ zeocin and incubated at 37°C overnight. DNA was extracted using the Qiagen Miniprep kit and submitted for sequencing, which confirmed the sequences of each gene.

### 2.2 Transformation of *K. phaffii* X-33 with *cbcas, thcas* or *cbdas*

Electrocompetent *K. phaffii* (strain X-33) cells were generated following an in-house lithium acetate method. A single colony was used to inoculate 10 mL Yeast Extract-Peptone-Dextrose medium and incubated at 30°C for 6 h with rotation at 180 rpm. The starter culture was used to inoculate 100 mL fresh YPD and grown as above overnight. Once the OD600 reached 1-2, cells were harvested by centrifugation at 5000 *g*, for 5 min at 4°C. The pellet was resuspended and incubated for 30 min in 25 mL of a solution containing 0.6 M sorbitol, 0.1 M lithium acetate, 10 mM DTT, 10 mM TrisHCl, pH 7.5. Occasional mixing was used to keep the cells in suspension. The cells were washed thrice using centrifugation as above but resuspended and incubated for 5 min in a solution containing 25 mL 1 M sorbitol at 0°C. Electrocompetent cells were then resuspended in 0.5 mL 1 M sorbitol at 0°C, and divided into 80 μL aliquots. All vectors were linearised using digestion with PmeI (New England Biolabs) and purified using a QIAquick Gel Extraction Kit (Qiagen). 2 μg of linearised vector was added to the aliquoted electrocompetent cells in a 2 mm electro-cuvette and incubated for 5 min on ice. Electroporation was performed on a Gene Pulser® II with a Capacitance Extender Plus and a Pulse Controller Plus (BioRad) set to 1.5 kV, capacitance 25 μF and resistance of 400 Ω, with pulses lasting 8-9 ms. 1 mL of a solution of 50:50 1 M sorbitol: YPD at 0°C was added immediately and the suspensions incubated for 4 h at 30°C with rotation at 800 rpm. The recombinant cells were then spread onto YPDZ-agar plates (YPD with zeocin at 100 μg mL^−1^), and incubated for 3 d at 30°C. The selected clones were subject to further colony purification by re-streaking on fresh YPDZ-agar plates, and incubated for 3 d at 30°C.

Clones displaying expression of the CSases were isolated by small scale test expressions. The colonies were used to inoculate 5 mL BMGY and incubated overnight at 30°C with shaking at 230 rpm. These starter cultures were used to inoculate 5 mL BMMY, supplemented with 10 mg L^−1^ riboflavin and 40 g L^−1^ bacto-casamino acids (mBMMY). The initial MeOH concentration was 1%. A 250 μL sample was taken at t = 0 h, with cell free broth obtained by centrifugation at 16,300*g* for 1 min. Every 24 h, a 250 μL timepoint sample was taken, with cell free broth obtained as above. To maintain the MeOH concentration, 250 μL mBMMY supplemented with 20% MeOH was added. The samples were analyzed by anti-His_6_ (Sigma) western blotting over the course of the induction (7 d).

### 2.3 Rich media fermentations

Fermentations were carried out using a 7-L, non-jacketed glass vessel, with the fermentation controlled by a Livit Flex and parameters plotted with Lucullus PIMS. The vessel was charged with 4 L mBMMY containing 100 μg L^−1^ ampicillin and 1% (v/v) MeOH. The temperature was maintained at 20°C throughout. The dissolved oxygen setpoint was set to 35%, which was maintained with a stirring cascade at 500-1000 rpm, with additional O_2_ gas supplied when required. A constant air supply of 0.5 L min^−1^ was supplied. The pH of the fermentation was set to 6.0, and maintained through the addition of 28% NH_4_OH, which also provided a nitrogen source.

Glycerol stocks of the selected clones were used to inoculate 5 mL YPD supplemented with 100 μg mL^−1^ zeocin and incubated overnight at 30°C with shaking at 180 rpm. The overnight culture was used to inoculate 500 mL YPD, supplemented with 100 μg mL^−1^ ampicillin and incubated as above, for 24 h. Cells were harvested by centrifugation at 5000 *g* for 5 min under sterile conditions and the pellets were resuspended in sterile distilled water (dH_2_O). The resuspended material was used to inoculate the bioreactor. Once the MeOH batch phase had ended, as indicated by a dissolved oxygen (dO_2_) spike, MeOH was added to a final concentration of 1% v/v. After ∼5 h, when the second batch phase had ended, MeOH feeding was conducted at a rate of 1.2 mL h^−1^ L ^−1^ overnight. The MeOH used for feeding was supplemented with 2.4 mL L^−1^ PTM_1_ salts. The feeding rate was incrementally increased to a maximum rate of 4.8 mL h^−1^ L ^−1^ over the course of the fermentation using dO_2_ spikes to assess if MeOH feed rate was limiting. The MeOH induction phase lasted for 168 h.

### 2.4 Purification and deglycosylation of the CSases

Cell free broth was subject to tangential flow diafiltration (Krosflo KR2i, MINIKROS sampler 10 kDa MWCO, 2600 cm^2^) to concentrate the material to ∼500 mL. The concentrate was buffer-exchanged into 50 mM sodium phosphate buffer at pH 7.4, containing 300 mM NaCl and 30 mM imidazole (His-Buffer A) by washing the concentrate with 4 vol of His buffer A. The buffer exchanged concentrate was then loaded onto a 5 mL Histrap™ FF Crude column, attached to an NGC ™ Discover™ plus (Biorad) at a rate of 3 mL min^−1^. The column was washed with 15 column volumes (CV) of His Buffer A before being eluted with 5 CV His buffer B (same as His buffer A but containing 500 mM imidazole). The peak fractions were diluted 10-fold with 50 mM sodium citrate buffer at pH 4.7 (CIEX buffer A) and loaded onto a 5 mL HiTrap™ SPHP column at a rate of 3 mL min^−1^. The column was washed with 10 CV CIEX buffer A, before being eluted over a 15 CV gradient between 0 and 100% CIEX buffer B (Same as CIEX A, but containing 1 M NaCl). Peak fractions were analyzed by SDS-PAGE. Fractions containing CSases were pooled and centrifugally concentrated (vivaspin turbo 10 kDA MWCO) to volumes of approximately 1 mL. The concentrate was loaded onto a Highload® Superdex®S75 16/600 column that was pre-equilibrated with 50 mM sodium citrate buffer at pH 4.7 containing 300 mM NaCl (SEC buffer A). The peak fractions were analyzed by SDS-PAGE (Figs. S2–S4).

The peak fractions containing the CSases were concentrated as above to a volume of approximately 1.5 mL. 0.1 mg Endo F1 (made in-house) and 200 μL Glyco buffer 3 (NEB) were added. The final volume was brought to 2 mL by addition of SEC buffer A. The de-glycosylation reaction was incubated overnight at 20°C with 500 rpm shaking. The mixture was injected onto a Highload® Superdex®S75 16/600 column that was pre-equilibrated with 50 mM HEPES, pH 7.0, 150 mM NaCl (SEC buffer B). Peak fractions were analyzed by SDS-PAGE. Fractions containing the CSases were pooled and centrifugally concentrated as above. Protein concentration was calculated using a BSA calibrated (0.125 – 2 mg mL^−1^) Bradford assay.

### 2.5 Crystallisation

Initial condition screening was performed using a 96 well sitting drop format, with a drop size of 300 nL with a 1:1 (protein:precipitant) drop ratio. Screens were decanted by Oryx8 (Douglas Instruments) using the commercially available INDEX (Hampton research) and PACT premier (Molecular dimensions). Crystallisation screens were incubated at 18-20°C with 25% humidity.

For the crystallisation of CBCAS, Endo H deglycosylated protein was used. Protein was concentrated to 20 mg mL^−1^ in 50 mM HEPES buffer at pH 7.0 containing 150 mM NaCl. Crystals grew over ∼2 weeks in INDEX condition B8 (1.4 M Sodium phosphate monobasic monohydrate/potassium phosphate dibasic, pH 8.2). Crystals were harvested into liquid nitrogen using mother liquor supplemented with 10% (v/v) ethylene glycol.

For crystallisation of the His-CBDAS, deglycosylated enzyme was concentrated to 35 mg mL^−1^ and incubated with 2 mM CBGA (dissolved in DMSO) for 30 min on ice. Precipitant material was removed by centrifugation at 16,300*g* for 5 min. Over ∼4 weeks, several hits were observed, again specifically in conditions D9 and F7-F12 in the INDEX Screen (Hampton Research). The diffracting crystal was grown from condition D9 (0.1 M Tris pH 8.5, 25% (w/v) Polyethylene glycol 3350) The crystal was harvested into liquid nitrogen with no additional cryoprotectant.

For the crystallisation of THCAS, deglycosylated enzyme was concentrated to 35 mg mL^−1^ and incubated with 2 mM CBGA (dissolved in DMSO) for 30 min on ice. Precipitant material was removed by centrifugation at 16,300*g* for 5 min. Initial screening was conducted using the PACT and INDEX screens (Hampton Research). The diffracting crystal grew over ∼3 weeks in INDEX condition D6 (0.1 M BIS-TRIS pH 5.5, 25% (w/v) Polyethylene glycol 3350). The crystal was harvested into liquid nitrogen with no additional cryoprotectant.

### 2.6 Data collection and refinement

Data for CBCAS, CBDAS and THCAS crystals were collected at the Diamond Light Source, Didcot, Oxfordshire, U.K. on beamline I03. Diffraction data were processed using Xia2 (Winter, 2010) and scaled and merged with AIMLESS (Evans and Murshudov, 2013). Data collection statistics can be found in Table S1. The datasets were obtained in space groups *P*42_1_2, *P*2_1_2_1_2_1_ and *P*22_1_2_1_ for CBCAS, CBDAS and THCAS respectively. Matthews coefficients for CBCAS, CBDAS and THCAS datasets were 2.97, 2.36 and 2.26, with 59%, 48% and 46% solvent content respectively for each crystal. Phasing for both CBDAS crystals and CBCAS was conducted using MOLREP (Vagin and Teplyakov, 1997), using AlphaFold2 (Jumper et al., 2021) *in silico* models for CBCAS and CBDAS and THCAS structure 3VTE (Shoyama et al., 2012) for THCAS. The structures were built and refined through iterative cycles of COOT (Emsley et al., 2010) and REFMAC (Murshudov et al., 1997). Refinement statistics can be found in Table S1. Coordinate and structure factor files for CBCAS and CBDAS and THCAS have been deposited in the Protein DataBank (PDB) with accession codes **29VZ**, **29WA** and **29VY** respectively.

## 3 Results and discussion

### 3.1 Cloning, expression and purification

In attempting to determine the structures of all three cannabinoid synthases, it was first necessary to choose appropriate sequences for gene cloning. The sequences chosen are listed in the Supporting Information (SI Section 1) and are those that arise from genome sequencing of the *Cannabis sativa*, with GenBank accession numbers for CBCAS, CBDAS and THCAS of AB057805.1, AB212831.1 and XM_030624886.1 and AB057805.1 respectively. The sequence of THCAS was identical to that presented by Shoyama and co-workers (Shoyama et al., 2012), although interestingly, the sequence of CBCAS featured a tryptophan residue at position 443, not the cysteine reported by Kayser and co-workers (Thomas and Kayser, 2023), which was mutated by that group to tryptophan to yield a CBCAS of improved activity for CBCA production. The genes, *cbcas*, *cbdas*, and *thcas*, which contained no introns, were synthesised with codon optimization for *Komagataella phaffii*, amplified by PCR and cloned into plasmid pPICZα-B, containing an N-terminal His-tag after the α-mating signal peptide, for the purposes of expression in *K*. *phaffii* using methanol induction. Linearised plasmids were used to transform the host using X-33 as described in the Materials and Methods section. Strains of *K. phaffii* carrying the recombinant genes for CBCAS, CBDAS and THCAS were grown in high cell density fed-batch fermentations at 20°C using containing rich media supplemented with PTM1 salts. Fermentation with typical basal salt media afforded limited yields of enzymes of crystallisation grade purity. Fermentations used a MeOH batch phase, followed by a MeOH fed batch phase as described in the Materials and Methods. For purification, the secretates from fermentation were first subjected to tangential flow, to reduce the volume sufficiently for the chromatographic steps. Each enzyme was purified using a sequence of nickel affinity chromatography followed by cation exchange and size exclusion chromatography (SI Section 2); the last step to exclude non-specific aggregates that would be detrimental to crystallisation. Crystallisation of the enzymes was achieved using each of the enzymes following deglycosylation with EndoH (CBCAS) or EndoF1 (CBDAS, THCAS) using methods described in the Materials and Methods section.

### 3.2 Overall structure of CSases

**Fig. 2:**
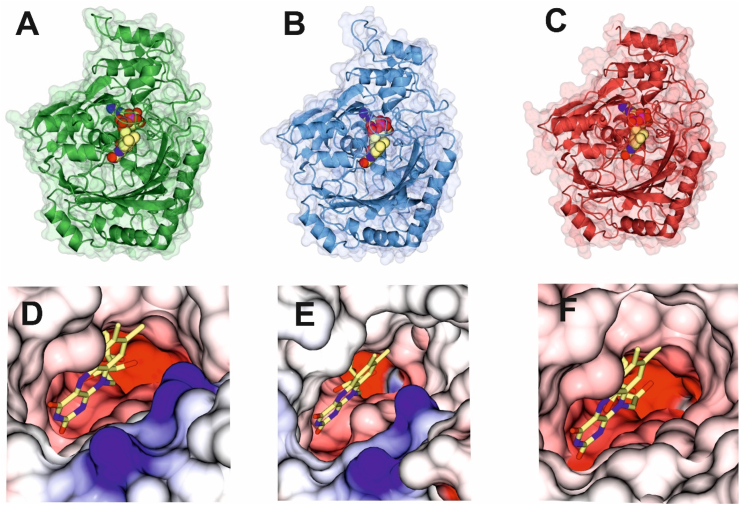
Structures of Enzymes **A**: CBCAS; **B**: CBDAS; **C**; THCAS. In each case the FAD is shown in sphere format with carbon atoms in yellow; **D, E**, **F**: access tunnels to active sites of CBCAS, CBDAS and THCAS respectively with electrostatic surfaces shown and FAD molecules in cylinder format with carbon atoms in yellow.

The structure of CBCAS was obtained in space-group *P*42_1_2 with one molecule in the asymmetric unit, and refined to a resolution of 2.43 Å (Fig. 2A; Data Collection and refinement Statistics can be found in Table S1 in the SI). There was electron density for almost all of the protein backbone between residues P30 and R543, except for regions T^299^DNHG and N^358^YNTA, which could not be modelled. CBCAS is a member of the BBE family of enzymes (Daniel et al., 2017), with an FAD binding domain (residues N29-V251 and 477-544) and a substrate binding domain (residues P252-N476). The omit maps clearly indicated the presence of FAD and continuous density between the side chains of H114 and C176 with the C8 and C6 carbon atoms of the FAD confirmed the bis-covalent attachment of the coenzyme to the protein. As the structure was obtained using protein following EndoH-catalyzed glycosylation, residual *N-*acetyl glucosamine residues were observed at residues N65, N89 and N499.

The structure of CBDAS (Fig. 2B) was obtained in space-group *P*22_1_2_1_, also with one molecule in the asymmetric unit, and refined to a resolution of 1.70 Å. As with CBCAS, almost all of the protein backbone and side-chains from P30 to R543 could be modelled except for N^358^YNTA. As for CBCAS, the omit maps again clearly showed the FAD and the bis-covalent attachment common to all three synthases. Residual *N-*acetyl glucosamine residues were observed at residues N65 and N498.

The structure of THCAS (Fig. 2C) was obtained in space-group *P*2_1_2_1_2_1_, but with three molecules in the asymmetric unit, and refined to a resolution of 2.33 Å, which was higher than that previously obtained by Shoyama and co-workers (3VTE) (Shoyama et al., 2012). Subunits B and C exhibited the best density, with main chain *B* factors for the three chains A, B and C of 61, 44 and 49 respectively. However, in common with 3VTE, while most of the protein backbone and side-chains from N29 to P541 could be modelled, poorer density was observed in approximately regions N^45^VAN, G^356^VVNFNTAN, A^375^G and K^491^INHASPNN. However, region D^300^NHGKNK, absent in 3VTE, could be modelled in the new data. Once again, the omit maps again clearly showed the FAD and the bis-covalent attachment to H114 and C176. Residual *N-*acetyl glucosamine residues were observed at residues N65, N89 and N305. Although both co-crystallisation and soaking experiments with CSase substrate CBGA **1** were conducted, no datasets for any enzyme featured density that could be successfully modelled as this ligand.

**Fig. 3:**
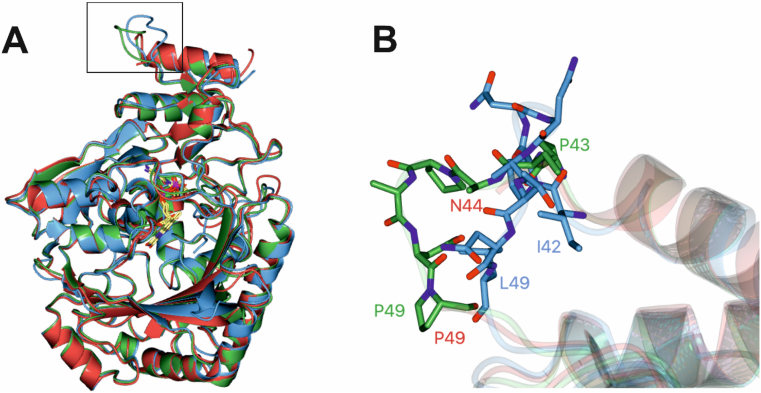
**A**: Superimposed structures of CBCAS, CBDAS and THCAS in ribbon format with backbones shown in green, blue and red respectively; **B**: Detail of loop in box corresponding to region N44-P49 (THCAS numbering) and showing difference between loops in CBCAS and CBDAS (carbon atoms in green and blue respectively).

The structures of CBCAS, CBDAS and THCAS were superimposed (Fig. 3A) to calculate rmsds (Table 2). The structures of CBCAS and THCAS were most similar, and those of CBDAS and THCAS most diverged, although rmsd values were small, indicative of high structural homology as would be expected, given the sequence homology of the three synthases.

**Table 2:** Sequence identity (%) and rmsds (Å over no. of Cα atoms) of the cannabinoid synthases.

	CBCAS	CBDAS	THCAS
**CBCAS**	100%; 0.00 (504)	82%; 0.80 (487)	93%; 0.65 (492)
**CBDAS**	82%; 0.80 (487)	100% 0.00 (497)	84%; 0.95 (485)
**THCAS**	93%; 0.65 (492)	84%; 0.95 (485)	100%; 0.00 (499)

One major difference (SI Figure S1) is the deletion of a serine residue S253 (THCAS) from CBDAS, which in THCAS/CBCAS is remote from the active site at the periphery of the enzyme and makes a H-bonding interaction with the side chain of N168. This explains the discrepancy in residue numbering between CBDAS, CBCAS and THCAS. The only notable differences in secondary structure are a relative displacement of loops N44-P49 (THCAS numbering**)** especially between CBCAS and CBDAS (Fig. 3B), and also N297 to T307. Other differences between CBDAS and CBCAS/THCAS include short runs of amino acids: N^44^N (CBDAS numbering) replaced by HD (CBCAS/THCAS) at the periphery of the structure although these are not additional glycosylation sites in CBDAS; L^171^AA for FPG in CBCAS/THCAS, in which the F introduces a new hydrophobic interaction with F185; R^342^QL for KEL(F), again at the periphery of the structures; S^394^VF for TAM; a hydrophobic interaction between F396 and V272 is replaced by M397 with F273 at this point; I^445^CS for TAT(S); C446 in CBDAS participates in an H-bond interaction with E406; I^490^NDPKN for KTNPES/KTNHAS in CBCAS and THCAS respectively; N491 participates in a H-bonding interactions with W405 in CBDAS; an interaction absent in both CBCAS and THCAS.

Taking CBCAS as a model, the closest structural homologs in the databases, as determined by the DALI server (Holm, 2022), and not including 3VTE, were a BBE-like enzyme from *Arabidopsis thaliana* (PDB code 5D79 (Daniel et al., 2016); Z value of 55.0; 48% sequence id, rmsd 1.6 Å over 482 Cα atoms); Bermuda grass isoallergen BG60 from *Cynodon dactylon* (PDB code 4DNS (Huang et al., 2012) 54.6; 47%, rmsd 1.4 Å over 480 Cα atoms) and reticuline oxidase from *Eschscholzia californica* (PDB code 3GSY (Winkler et al., 2009), 53.8; 41%, rmsd 1.5 Å over 484 Cα atoms); Major structural differences between CBCAS and 3GSY included extended loop regions in the former at R296-T307 (3GSY: D286-Q289) and I42-F51 (N34-N38) but a shorter loop at Q55-L59 (F42-D49). The active site residues of the CSases and reticuline oxidase were substantially different as might be expected given their divergent reaction chemistries.

### 3.3 Active sites of CBCAS, CBDAS and THCAS

**Fig. 4:**
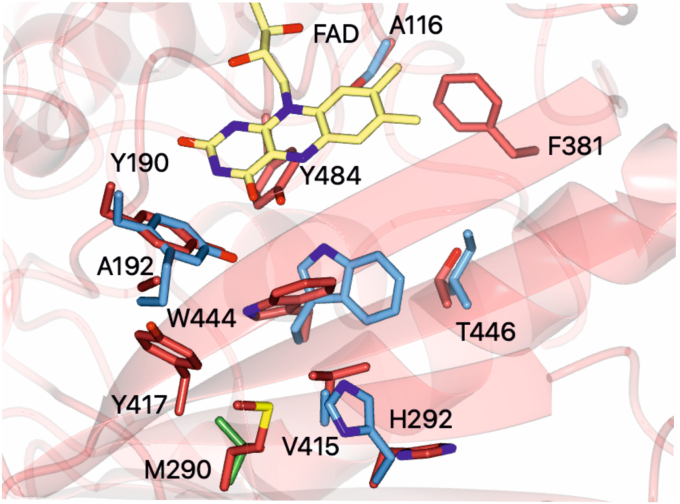
Superposition of active sites of CBCAS, CBDAS and THCAS with carbon atoms of side chains shown in green, blue and red respectively. Backbone in shade, conserved residues that exhibit little change in all three structures, and FAD (C atoms in yellow) are all those of THCAS with THCAS numbering, presented for context.

The structural similarities of the global folds of the three synthases are at odds with their distinct reaction specificities. It is useful therefore to summarise the differences between the active sites of the enzymes, wherein the control of chemoselectivity is most likely to be exerted. A superimposition of the active sites is shown in Fig. 4.

Several residues are conserved among all three CSases, including many that have been proposed to have roles in substrate binding or catalysis. Conserved active site residues include Y483/4 (CBDAS/CBCAS and THCAS numbering), W443/4, Y190 and H291/2. The position of Y483/4, which has been proposed to act as a general base in THCAS catalysis for deprotonation of the hydroxyl group in the 1-position of the aromatic ring (Shoyama et al., 2012) (proposed mechanisms of CSase catalysis, adapted from Tahir and co-workers (Tahir et al., 2021), are shown in Fig. S5), was largely unchanged in the three enzyme structures. The first difference in the active site between THCAS/CBCAS and CBDAS is that in the latter W443 (W444) is markedly rotated towards the substrate binding pocket beneath the flavin. Close examination of the active sites reveals that this displacement is most likely due to Y190 impinging on the alternative position found in THCAS/CBCAS; the slight movement of Y190 is caused by the greater rigidity of the loop bearing this residue featuring P192 in CBDAS rather than an alanine as in THCAS/CBCAS. The side-chain of H291/2, which has been proposed to stabilise CBGA in the active site of CBDAS/CBCAS/THCAS by neutralising the carboxylate anion of CBGA (Shoyama et al., 2012), is found in the same conformation in CBCAS and CBDAS, but in THCAS is rotated away from the FAD towards the active site channel.

The structures also reveal the active site context of non-conserved residues, most interestingly those that distinguish CBDAS from the more closely related CBCAS and THCAS. Some of these changes substantially alter the topology of the active site of CBDAS commensurate with its role in catalysing a chemoselective reaction distinct from either CBCAS or THCAS, which each form a benzopyran ring in their products. Near the FAD, S116 in CBDAS is substituted for alanine in THCAS/CBCAS, although this residue is not close to the probable site of substrate binding. A414 in CBDAS is substituted for valine 415 in THCAS/CBCAS. The presence of this smaller residue makes space to permit the rotation of W443 in the CBDAS active site described above. I445 in CBDAS is substituted for threonine 446 in THCAS/CBCAS. This isoleucine forms a hydrophobic interaction with the rotated W443; an interaction that would not occur with W444 in the CBCAS/THCAS conformation. Further differences are present away from the FAD and lining the active site channel and entrance, where two differences between CBCAS and CBDAS/THCAS are observed. CBDAS/THCAS M289/290 is replaced by the shorter, polar T290 in CBCAS and I293/294 in CBDAS/THCAS is replaced by an arginine R294 in CBCAS.

The acquisition of the three CSase structures may prompt the creation and analysis of models that are docked with substrate CBGA **1**, in order to speculate on how different active site architectures may result in the different enzyme chemoselectivities. However, the flexibility of the substrate in modelling experiments means that we have found such modelling to be uninformative, with many different substrate poses with equivalent energies obtained. The structure of the CSases reported above do nevertheless provide a molecular context for the effects of some of the mutational studies (Zirpel et al., 2018; Thomas and Kayser, 2023; Dai et al., 2024; Qi et al., 2025; Shoyama et al., 2012) and natural variant analyses (Onofri et al., 2015) performed by other groups. The specific contribution of individual amino acids to catalysis remains cryptic however in the absence of ligand complexes that show the interactions between the active site and the common CSase substrate CBGA.

## 4 Conclusion

The biotechnological preparation of natural and non-natural cannabinoids has great potential if more selective biocatalysts can be designed for the synthesis of the most important compounds. The engineering of the cannabinoid synthases for improved selectivity will benefit from improved protocols for heterologous expression, mutational studies, modelling, and also structural information that provides detailed information on the differences between the active sites. Although we have been unable to determine the structures of the synthases in complex with CBGA, the FAD complexes presented should provide a robust platform for better informed modelling and further engineering studies that look to alter and improve the enzymes for process applications.

## Credit author statement

A.K., E.B., J.C. and G.G. were responsible for conceptualization and acquiring funding. J.D. was responsible for Methodology and Data curation. All authors were responsible for analysis of the results and visualization; J.D. and G.G. wrote the original draft, which was also reviewed and approved by all authors.

## Funding

This work was funded by 10.13039/100011096Jazz Pharmaceuticals.

## Declaration of competing interest

The authors declare the following financial interests/personal relationships which may be considered as potential competing interests: Jack Domenech reports financial support was provided by Jazz Pharmaceuticals UK Limited. If there are other authors, they declare that they have no known competing financial interests or personal relationships that could have appeared to influence the work reported in this paper.
