# Supplementary material for: X-ray crystal structures of the cannabinoid synthases CBCAS, CBDAS and THCAS
Source: Curr Res Struct Biol. 2026 Jul 16;12:100197. doi: 10.1016/j.crstbi.2026.100197 (PMC13400433; doi:10.1016/j.crstbi.2026.100197)
Supplement: Multimedia component 1 [file mmc1.pdf]

X-ray crystal structures of the cannabinoid synthases CBCAS, CBDAS and THCAS

Jack Domenech,[a] Andrew King,[b] Edward Byrne,[b] Jared Cartwright,[c] and Gideon Grogan[a*]*

[a]Department of Chemistry, University of York, Heslington, York YO10 5DD UK

[b]Jazz Pharmaceuticals, Kent Science Park, Sittingbourne ME8 9AG UK

[c]Department of Biology, University of York, Heslington, York YO10 5DD UK

SUPPORTING INFORMATION

Table of Contents

Section 1. Sequences of Enzymes used for Cloning and Expression in *P. pastoris* S3

Section 2. Purification and deglycosylation of the CSases S6

Section 3. Data Collection and Refinement S8

Section 4. Previously published mechanistic proposal S10

Section 1. Sequences of Enzymes used for Cloning and Expression in *P. pastoris*

>N-His CBCAS

MRFPSIFTAVLFAASSALAAPVNTTTEDETAQIPAEAVIGYSDLEGDFDVAVLPFSNSTNNG

LLFINTTIASIAAKEEGVSLEKRGSSHHHHHHNPQENFLKCFSEYIPNNPANPKFIYTQHDQ

LYMSVLNSTIQNLRFTSDTTPKPLVIVTPSNVSHIQASILCSKKVGLQIRTRSGGHDAEGLS

YISQVPFAIVDLRNMHTVKVDIHSQTAWVEAGATLGEVYYWINEMNENFSFPGGYCPTVGVG

GHFSGGGYGALMRNYGLAADNIIDAHLVNVDGKVLDRKSMGEDLFWAIRGGGGENFGIIAAW

KIKLVVVPSKATIFSVKKNMEIHGLVKLFNKWQNIAYKYDKDLMLTTHFRTRNITDNHGKNK

TTVHGYFSSIFLGGVDSLVDLMNKSFPELGIKKTDCKELSWIDTTIFYSGVVNYNTANFKKE

ILLDRSAGKKTAFSIKLDYVKKLIPETAMVKILEKLYEEEVGVGMYVLYPYGGIMDEISESA

IPFPHRAGIMYELWYTATWEKQEDNEKHINWVRSVYNFTTPYVSQNPRLAYLNYRDLDLGKT

NPESPNNYTQARIWGEKYFGKNFNRLVKVKTKADPNNFFRNEQSIPPLPPRHH

>N-His CBDAS

MRFPSIFTAVLFAASSALAAPVNTTTEDETAQIPAEAVIGYSDLEGDFDVAVLPFSNSTNNG

LLFINTTIASIAAKEEGVSLEKRGSSHHHHHHNPRENFLKCFSQYIPNNATNLKLVYTQNNP

LYMSVLNSTIHNLRFTSDTTPKPLVIVTPSHVSHIQGTILCSKKVGLQIRTRSGGHDSEGMS

YISQVPFVIVDLRNMRSIKIDVHSQTAWVEAGATLGEVYYWVNEKNENLSLAAGYCPTVCAG

GHFGGGGYGPLMRNYGLAADNIIDAHLVNVHGKVLDRKSMGEDLFWALRGGGAESFGIIVAW

KIRLVAVPKSTMFSVKKIMEIHELVKLVNKWQNIAYKYDKDLLLMTHFITRNITDNQGKNKT

AIHTYFSSVFLGGVDSLVDLMNKSFPELGIKKTDCRQLSWIDTIIFYSGVVNYDTDNFNKEI

LLDRSAGQNGAFKIKLDYVKKPIPESVFVQILEKLYEEDIGAGMYALYPYGGIMDEISESAI

PFPHRAGILYELWYICSWEKQEDNEKHLNWIRNIYNFMTPYVSKNPRLAYLNYRDLDIGIND

PKNPNNYTQARIWGEKYFGKNFDRLVKVKTLVDPNNFFRNEQSIPPLPRHRH

>N-His THCAS

MRFPSIFTAVLFAASSALAAPVNTTTEDETAQIPAEAVIGYSDLEGDFDVAVLPFSNSTNNG

LLFINTTIASIAAKEEGVSLEKRGSSHHHHHHNPRENFLKCFSKHIPNNVANPKLVYTQHDQ

LYMSILNSTIQNLRFISDTTPKPLVIVTPSNNSHIQATILCSKKVGLQIRTRSGGHDAEGMS

YISQVPFVVVDLRNMHSIKIDVHSQTAWVEAGATLGEVYYWINEKNENLSFPGGYCPTVGVG

GHFSGGGYGALMRNYGLAADNIIDAHLVNVDGKVLDRKSMGEDLFWAIRGGGGENFGIIAAW

KIKLVAVPSKSTIFSVKKNMEIHGLVKLFNKWQNIAYKYDKDLVLMTHFITKNITDNHGKNK

TTVHGYFSSIFHGGVDSLVDLMNKSFPELGIKKTDCKEFSWIDTTIFYSGVVNFNTANFKKE

ILLDRSAGKKTAFSIKLDYVKKPIPETAMVKILEKLYEEDVGAGMYVLYPYGGIMEEISESA

IPFPHRAGIMYELWYTASWEKQEDNEKHINWVRSVYNFTTPYVSQNPRLAYLNYRDLDLGKT

NHASPNNYTQARIWGEKYFGKNFNRLVKVKTKVDPNNFFRNEQSIPPLPPHHH

For all N-His constructs, residues in bold denote the α-factor signal peptide from *S. cerevisiae*

```
CBCAS      NPQENFLKCFSEYIPNNPANPKFIYTQHDQLYMSVLNSTIQNLRFTSDTTPKPLVIVTPS
THCAS      NPRENFLKCFSKHIPNNVANPKLVYTQHDQLYMSILNSTIQNLRFISDTTPKPLVIVTPS
CBDAS      NPRENFLKCFSQYIPNNATNLKLVYTQNNPLYMSVLNSTIHNLRFTSDTTPKPLVIVTPS
           **:********::**** :* *::***:: ****:*****:**** **************

CBCAS      NVSHIQASILCSKKVGLQIRTRSGGHDAEGLSYISQVPFAIVDLRNMHTVKVDIHSQTAW
THCAS      NNSHIQATILCSKKVGLQIRTRSGGHDAEGMSYISQVPFVVVDLRNMHSIKIDVHSQTAW
CBDAS      HVSHIQGTILCSKKVGLQIRTRSGGHDSEGMSYISQVPFVIVDLRNMRSIKIDVHSQTAW
           : ****.:*******************:**:********.:******:::*:*:******

CBCAS      VEAGATLGEVYYWINEMNENFSFPGGYCPTVGVGGHFSGGGYGALMRNYGLAADNIIDAH
THCAS      VEAGATLGEVYYWINEKNENLSFPGGYCPTVGVGGHFSGGGYGALMRNYGLAADNIIDAH
CBDAS      VEAGATLGEVYYWVNEKNENLSLAAGYCPTVCAGGHFGGGGYGPLMRNYGLAADNIIDAH
           *************:** ***:*:..****** .****.*****.****************

CBCAS      LVNVDGKVLDRKSMGEDLFWAIRGGGGENFGIIAAWKIKLVVVPSKATIFSVKKNMEIHG
THCAS      LVNVDGKVLDRKSMGEDLFWAIRGGGGENFGIIAAWKIKLVAVPSKSTIFSVKKNMEIHG
CBDAS      LVNVHGKVLDRKSMGEDLFWALRGGGAESFGIIVAWKIRLVAVP-KSTMFSVKKIMEIHE
           ****.****************:****.*.****.****:**.** *:*:***** ****

CBCAS      LVKLFNKWQNIAYKYDKDLMLTTHFRTRNITDNHGKNKTTVHGYFSSIFLGGVDSLVDLM
THCAS      LVKLFNKWQNIAYKYDKDLVLMTHFITKNITDNHGKNKTTVHGYFSSIFHGGVDSLVDLM
CBDAS      LVKLVNKWQNIAYKYDKDLLLMTHFITRNITDNQGKNKTAIHTYFSSVFLGGVDSLVDLM
           ****.**************:* *** *:*****:*****::* ****:* **********

CBCAS      NKSFPELGIKKTDCKELSWIDTTIFYSGVVNYNTANFKKEILLDRSAGKKTAFSIKLDYV
THCAS      NKSFPELGIKKTDCKEFSWIDTTIFYSGVVNFNTANFKKEILLDRSAGKKTAFSIKLDYV
CBDAS      NKSFPELGIKKTDCRQLSWIDTIIFYSGVVNYDTDNFNKEILLDRSAGQNGAFKIKLDYV
           **************:::***** ********::* **:**********:: **.******

CBCAS      KKLIPETAMVKILEKLYEEEVGVGMYVLYPYGGIMDEISESAIPFPHRAGIMYELWYTAT
THCAS      KKPIPETAMVKILEKLYEEDVGAGMYVLYPYGGIMEEISESAIPFPHRAGIMYELWYTAS
CBDAS      KKPIPESVFVQILEKLYEEDIGAGMYALYPYGGIMDEISESAIPFPHRAGILYELWYICS
           ** ***:.:*:********::*.***.********:***************:***** .:

CBCAS      WEKQEDNEKHINWVRSVYNFTTPYVSQNPRLAYLNYRDLDLGKTNPESPNNYTQARIWGE
THCAS      WEKQEDNEKHINWVRSVYNFTTPYVSQNPRLAYLNYRDLDLGKTNHASPNNYTQARIWGE
CBDAS      WEKQEDNEKHLNWIRNIYNFMTPYVSKNPRLAYLNYRDLDIGINDPKNPNNYTQARIWGE
           **********:**:*.:*** *****:*************:* .:  .************

CBCAS      KYFGKNFNRLVKVKTKADPNNFFRNEQSIPPLPPRHH
THCAS      KYFGKNFNRLVKVKTKVDPNNFFRNEQSIPPLPPHHH
CBDAS      KYFGKNFDRLVKVKTLVDPNNFFRNEQSIPPLPRHRH
           *******:******* .**************** ::*
```

Figure S1. Sequence alignment of **CBCAS**, **CBDAS** and **THCAS**. Residues in **bold amber** are conserved between any two enzymes; those in **bold black underlined** are unique to one enzyme; residues in yellow boxes (**CBCAS** Y190, H292, F381, W444, Y484) are active site residues conserved between all three enzymes. Uniprot accession numbers for **CBCAS**: Q33DQ2 (no. of residues 545; MW 62039 Da; theoretical pI 8.85); **CBDAS**: A6P6V9 (544; 62237 Da; 8.88) and **THCAS**: Q8GTB6 (545; 61927 Da; 9.04).

Section 2. Purification and deglycosylation of the CSases

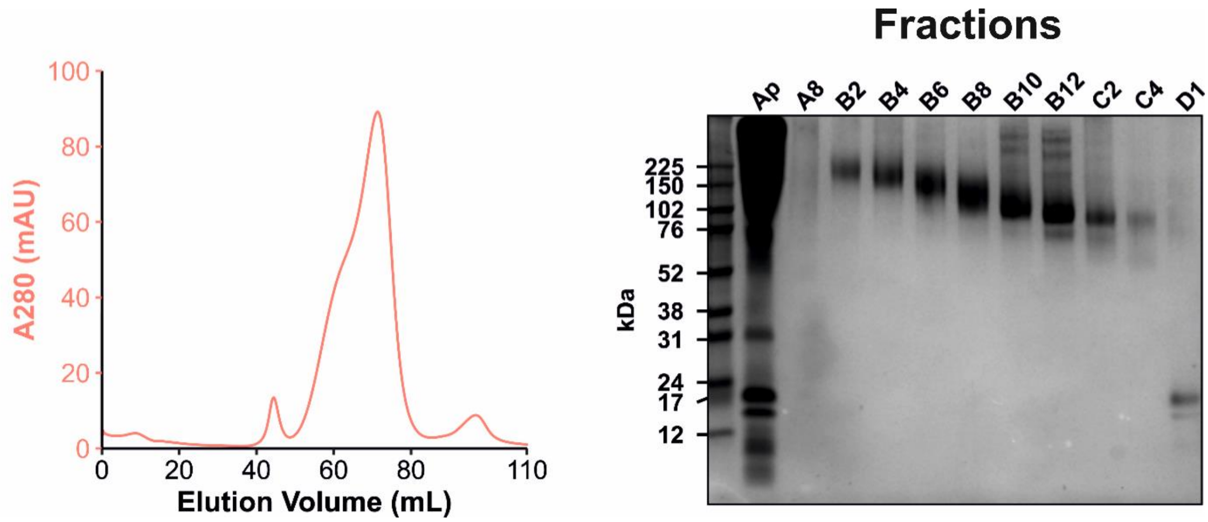

Figure S2: SEC purification of CBDAS from CIEX fractions. Left: SEC chromatogram monitoring eluent at 280 nm. Right: SDS-PAGE analysis of fractions: ‘Ap’ = material applied to column; A8 *etc.* refers to row and number of fraction in the fraction collector.

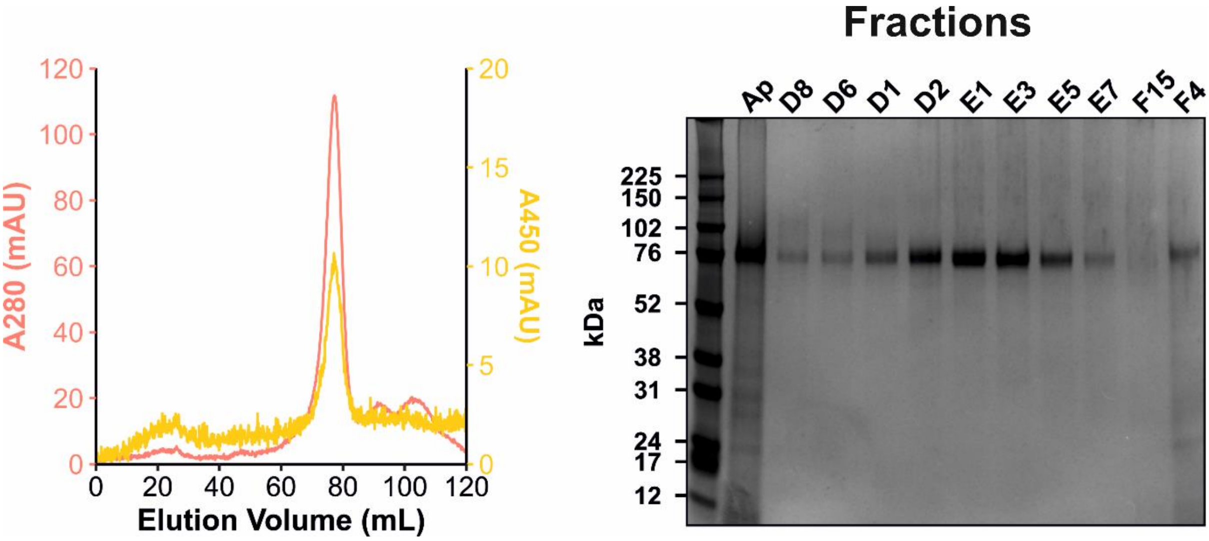

Figure S3. SEC purification of CBCAS from CIEX fractions. Left: SEC chromatogram monitoring eluent at 280 nm and 450 nm. Right: SDS-PAGE analysis of fractions. ‘Ap’ = material applied to column; D8 *etc.* refers to row and number of fraction in the fraction collector.

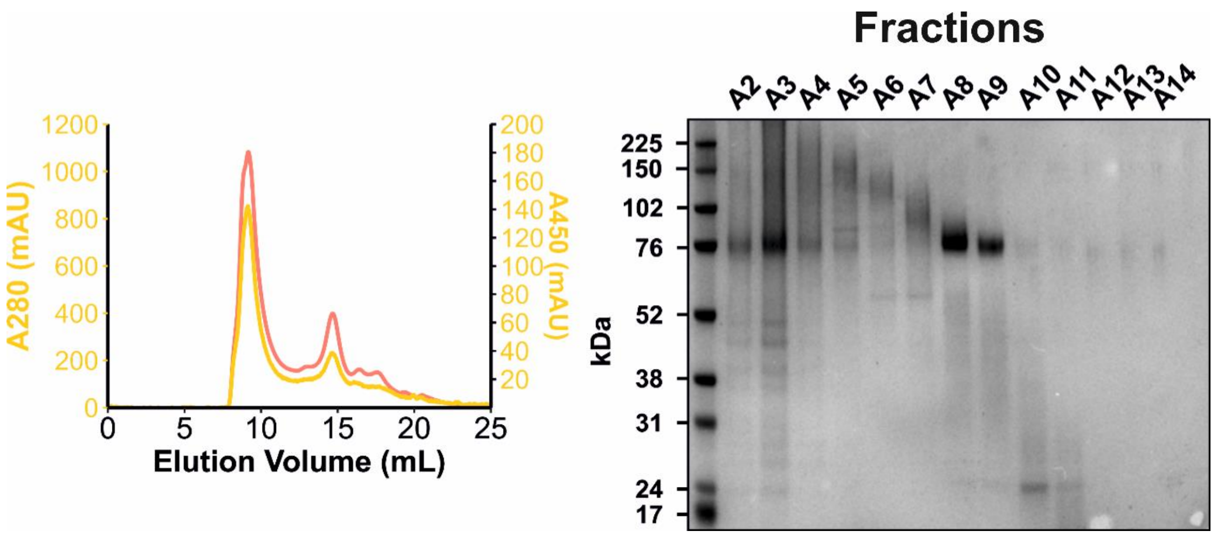

Figure S4: SEC purification of THCAS from CIEX fractions. Left: chromatography trace monitoring eluent at 280 nm and 450 nm. Right: SDS-PAGE analysis of fractions. A2 *etc.* refers to row and number of fraction in the fraction collector.

Section 3. Data Collection and Refinement

Table S1. Data statistics and refinement statistics for the CBCAS-FAD, CBDAS-FAD and THCAS-FAD datasets. Values in parentheses refer to data in the highest resolution shells.

	CBCAS-FAD	CBDAS-FAD	THCAS-FAD
Beamline	I03	I03	I03
Wavelength (Å)	0.9763	0.9763	0.9763
Resolution (Å)	66.68-2.43 (2.52-2.43)	59.34-1.70 (1.73-1.70)	58.97-2.33 (2.38-2.33)
Space Group	$P42_12$	$P22_12_1$	$P2_12_12_1$
Unit cell (Å)	a = 102.41; b = 102.41; c = 133.37 α = β = γ = 90.00°	a = 63.02; b = 66.00; c = 135.56 α = β = γ = 90.00°	a = 58.13; b = 134.93; c = 196.67 α = β = γ = 90.00°
No. of molecules in the asymmetric unit	1	1	3
Unique reflections	27448 (2818)	63060 (3273)	67180 (4425)
Completeness (%)	100.0 (100.0)	100.0 (100.0)	100.0 (100.0)
R_{merge} (%)	0.09 (1.41)	0.14 (1.43)	0.11 (0.75)
$R_{p.i.m.}$	0.03 (0.39)	0.06 (0.58)	0.04 (0.28)
Multiplicity	26.5 (27.3)	13.5 (13.7)	13.5 (13.6)
$<I/\sigma(I)>$	24.4 (3.1)	11.7 (1.9)	14.3 (3.2)
Overall *B* from Wilson plot (Å^2)	57	17	37
$CC_{1/2}$	1.00 (0.97)	1.00 (0.73)	1.00 (0.96)
R_{cryst}/ R_{free} (%)	0.20/0.26	0.15/0.18	0.25/0.30
r.m.s.d 1-2 bonds (Å)	0.008	0.010	0.007
r.m.s.d 1-3 angles (°)	1.760	1.821	1.640
Avge main chain B (Å^2)	70	19	51
Avge side chain B (Å^2)	74	23	53
Avge waters B (Å^2)	62	33	43
Avge Cofactor B (Å^2)	58	14	39

No. fit residues for each chain	480	493	1465
No protein atoms used in refinement	3946	3976	11329
Number of ligand atoms (FAD) used in refinement	51	51	153
Number of solvent atoms used in refinement	79	374	292
Ramachandran plot statistics	93.3% residues (favoured regions)<br><br>6.7% residues (allowed regions)	96.5% residues (favoured regions)<br><br>3.5% residues (allowed regions)	95.0% residues (favoured regions)<br><br>4.2% residues (allowed regions)<br><br>0.8% residues (disfavoured regions)

Section 4. Previously published mechanistic proposal

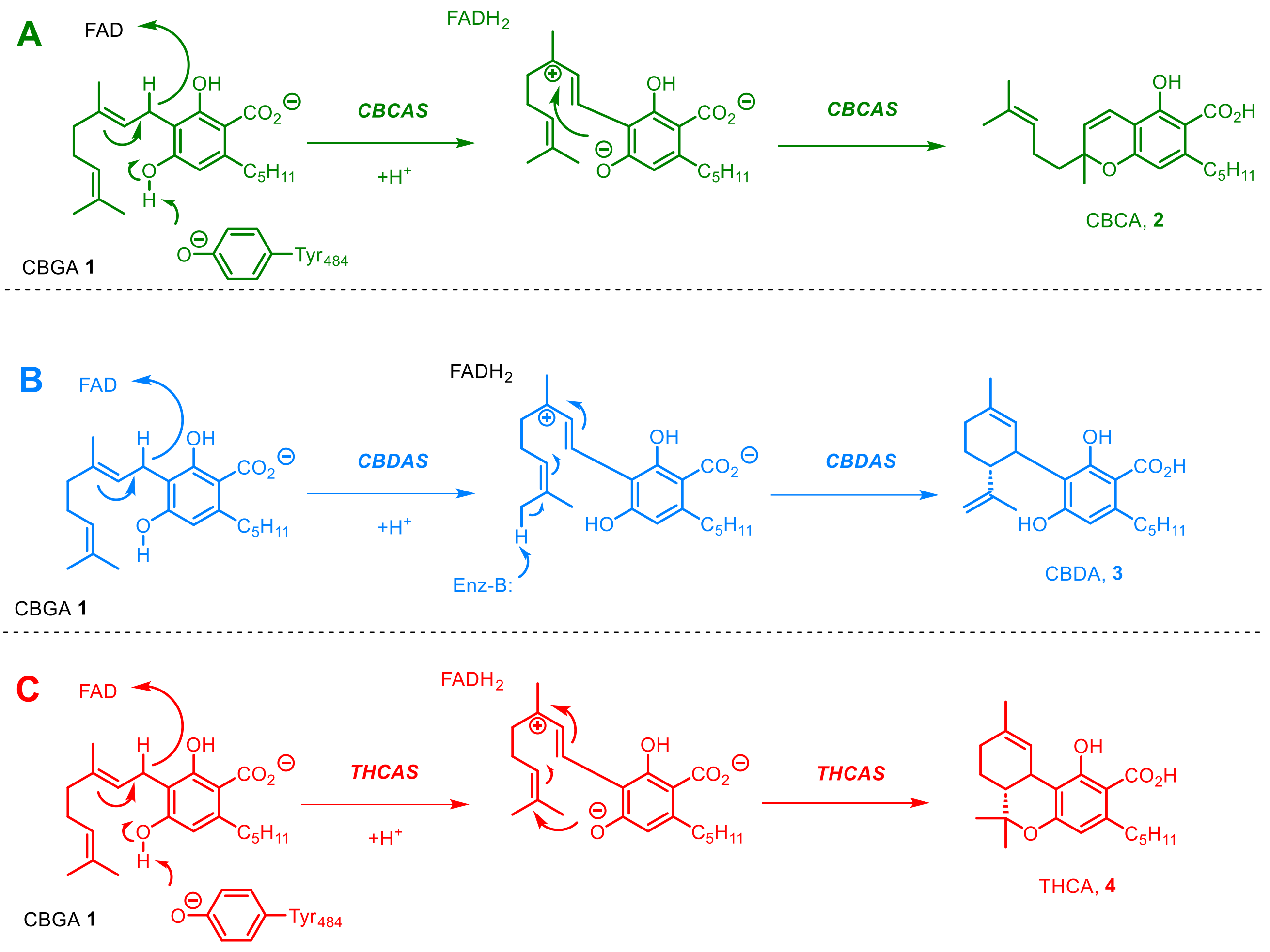

Figure S5. Suggested mechanisms for chemodivergent outcomes of cannabinoid synthase enzyme activity. **A**: CBCAS; **B**: CBDAS; **C**: THCAS (adapted from Tahir and co-workers [1]).

References

[1] M.N. Tahir, F.S. Raz, S. Rondeau-Gagné, J.F. Trant, 2021. The biosynthesis of the cannabinoids, J. Cannabis Res., 3, 7. https://doi.org/10.1186/s42238-021-00062-4.
